# 4-(4-Bromo­benzene­sulfonamido)benzoic acid

**DOI:** 10.1107/S1600536809013798

**Published:** 2009-04-18

**Authors:** Islam Ullah Khan, Ghulam Mustafa, Muhammad Nadeem Arshad, Muhammad Shafiq, Shahzad Sharif

**Affiliations:** a Materials Chemistry Laboratory, Department of Chemistry, Government College University, Lahore, Pakistan

## Abstract

The title compound, C_13_H_10_BrNO_4_S, belongs to the sulfonamide class of organic compounds. The two aromatic rings are inclined at 34.30 (15)° to one another, and the carboxyl substituent lies in the plane of the benzene ring to which it is bound (maximum deviation = 0.004 Å). In the crystal structure, charactersitic carboxylic acid dimers are formed through O—H⋯O hydrogen bonds. These dimers are linked into rows down *a* by N—H⋯O inter­actions. Additional C—H⋯O contacts further stabilize the structure, and a close Br⋯Br(*x*, −*y* + 1, −*z* + 1) contact of 3.5199 (9) Å is also observed.

## Related literature

For details of the biological activity and pharmaceutical applications of sulfonamide derivatives, see: Pandya *et al.* (2003 ▶); Supuran & Scozzafava (2000 ▶); Arshad, Khan & Zia-ur-Rehman (2008 ▶). For thia­zine-related heterocycles, see: Arshad, Tahir *et al.* (2008 ▶). For a related structure, see: Nan & Xing (2006 ▶). For bond-length information, see: Allen *et al.* (1987 ▶). For the synthesis, see: Deng & Mani (2006 ▶).
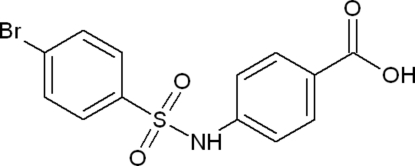


## Experimental

### 

#### Crystal data

C_13_H_10_BrNO_4_S
*M* *_r_* = 356.19
Monoclinic, 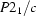

*a* = 5.1344 (5) Å
*b* = 13.1713 (11) Å
*c* = 20.0224 (19) Å
β = 91.730 (5)°
*V* = 1353.4 (2) Å^3^
*Z* = 4
Mo *K*α radiation
μ = 3.20 mm^−1^
*T* = 296 K
0.35 × 0.21 × 0.09 mm

#### Data collection

Bruker Kappa APEXII CCD diffractometer
Absorption correction: multi-scan (*SADABS*; Bruker, 2007 ▶) *T*_min_ = 0.448, *T*_max_ = 0.754
14856 measured reflections
3352 independent reflections
1838 reflections with *I* > 2σ(*I*)
*R*_int_ = 0.061

#### Refinement

*R*[*F* ^2^ > 2σ(*F* ^2^)] = 0.048
*wR*(*F* ^2^) = 0.128
*S* = 1.01
3352 reflections
182 parameters
H-atom parameters constrained
Δρ_max_ = 1.43 e Å^−3^
Δρ_min_ = −1.09 e Å^−3^

### 

Data collection: *APEX2* (Bruker, 2007 ▶); cell refinement: *SAINT* (Bruker, 2007 ▶); data reduction: *SAINT*; program(s) used to solve structure: *SHELXS97* (Sheldrick, 2008 ▶); program(s) used to refine structure: *SHELXL97* (Sheldrick, 2008 ▶); molecular graphics: *ORTEP-3 for Windows* (Farrugia, 1997 ▶) and *PLATON* (Spek, 2009 ▶); software used to prepare material for publication: *WinGX* (Farrugia, 1999 ▶) and *PLATON*.

## Supplementary Material

Crystal structure: contains datablocks I, global. DOI: 10.1107/S1600536809013798/sj2609sup1.cif

Structure factors: contains datablocks I. DOI: 10.1107/S1600536809013798/sj2609Isup2.hkl

Additional supplementary materials:  crystallographic information; 3D view; checkCIF report

## Figures and Tables

**Table 1:** Hydrogen-bond geometry (Å, °)

*D*—H⋯*A*	*D*—H	H⋯*A*	*D*⋯*A*	*D*—H⋯*A*
O2—H2*A*⋯O1^i^	0.82	1.80	2.606 (4)	171
N1—H1⋯O4^ii^	0.86	2.57	3.001 (3)	112
C2—H2⋯O1^iii^	0.93	2.46	3.361 (5)	164
C3—H3⋯O2^iv^	0.93	2.53	3.314 (5)	143
C11—H11⋯O3^v^	0.93	2.58	3.395 (5)	146

Symmetry codes: (i) 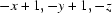; (ii) 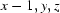; (iii) 
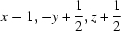
; (iv) 
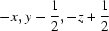
; (v) 
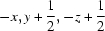
.

**Fig. 1.**
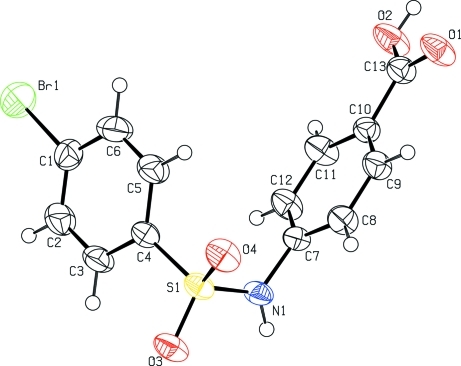
The structure of (I) with displacement ellipsoids for the non-hydrogenatoms drawn at the 50% probability level.

**Fig. 2.**
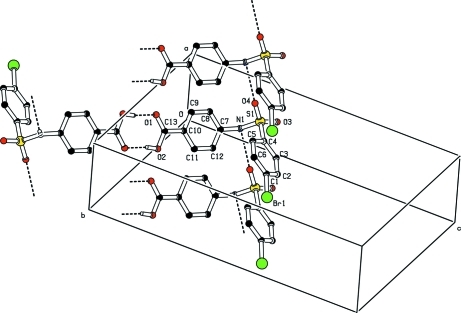
Crystal packing for (I) showing the formation of rows of dimers with hydrogen bonds drawn as dashed lines and H atoms not involved in hydrogen bonding omitted.

**Fig. 3.**
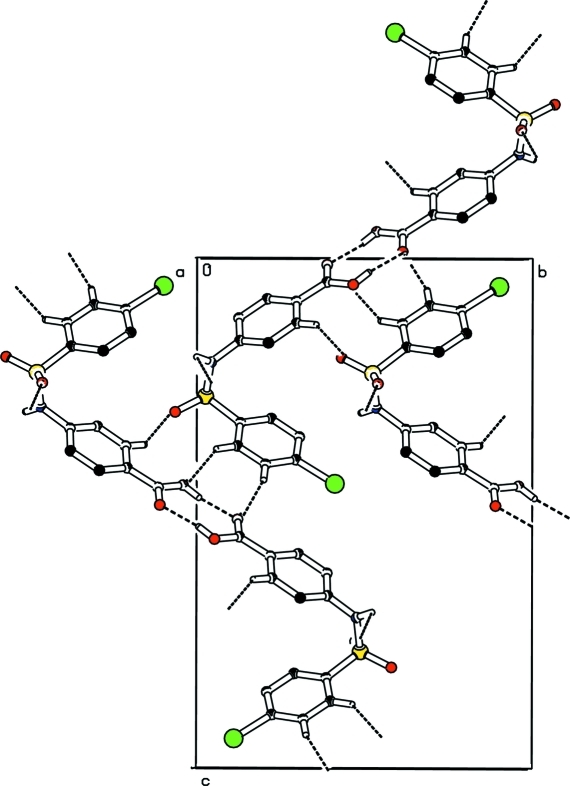
Unit cell packing for (I) showing additional C–H···O hydrogen bonds drawn as dashed lines and H atoms not involved in hydrogen bonding omitted.

## supplementary crystallographic information

### Comment

Sulfonamide derivatives have been reported as antibacterial agents (Pandya *et al.*, 2003) as well as enzyme inhibitors. The studies also revealed that aromatic sulfonamides are inhibitors of the growth of tumor cells (Supuran, & Scozzafava, 2000). Herein we report the structure of the title compound I, Fig, 1, as a continuation of our work on the synthesis and structure of sulfonamides (Arshad, Khan & Zia-ur-Rehman *et al.*, 2008*a*) and thiazine related heterocycles (Arshad, Tahir *et al.*, 2008*b*).

The structure of the title compound I can be compared with that of 4-(tosylamino)benzoic acid (Nan and Xing, 2006) which differs only in respect that I has bromo substituent in the *para* position instead of methyl group. The carboxylic acid substituent lies in the plane of the benzene ring to which it is bound (maximum deviation 0.004 Å) and the phenyl rings (C1—C6) and (C7—C12) are oriented at an angle of 34.30 (0.15) ° to each other. Bond lengths in the molecule are normal (Allen *et al.*, 1987). The carboxylic acid substituent forms dimers *via* intermolecular O—H···O hydrogen bonds. These dimers are further linked through N–H···O hydrogen bonds between the N–H and the oxygen of the sulfonyl group (SO_2_) along the *a* axis. Moreover the structure is further stabilized by C–H···O intermolecular interactions, Table 1, by forming seven and ten membered ring motifs Fig. 3.

### Experimental

The title compound was synthesized following the method (Deng & Mani, 2006). and recrystallized from ethanol for X-ray studies.

### Refinement

All H-atoms were positioned geometrically and refined using a riding model with d(C—H) = 0.93 Å, *U*_iso_=1.2*U*_eq_ (C) for aromatic 0.82 Å, *U*_iso_ = 1.5*U*_eq_ (O) for the OH group and 0.86 Å, *U*_iso_ = 1.2*U*_eq_ (N) for the NH group.

### Crystal data

**Table:**

C_13_H_10_BrNO_4_S	*F*(000) = 712
*M**_r_* = 356.19	*D*_x_ = 1.748 Mg m^−^^3^
Monoclinic, *P*2_1_/*c*	Mo *K*α radiation, λ = 0.71073 Å
Hall symbol: -P 2ybc 1	Cell parameters from 2704 reflections
*a* = 5.1344 (5) Å	θ = 2.6–22.0°
*b* = 13.1713 (11) Å	µ = 3.20 mm^−^^1^
*c* = 20.0224 (19) Å	*T* = 296 K
β = 91.730 (5)°	Irregular fragment, white
*V* = 1353.4 (2) Å^3^	0.35 × 0.21 × 0.09 mm
*Z* = 4	

### Data collection

**Table:**

Bruker Kappa APEXII CCD diffractometer	3352 independent reflections
Radiation source: fine-focus sealed tube	1838 reflections with *I* > 2σ(*I*)
graphite	*R*_int_ = 0.061
φ and ω scans	θ_max_ = 28.3°, θ_min_ = 2.6°
Absorption correction: multi-scan (*SADABS*; Bruker, 2007)	*h* = −6→6
*T*_min_ = 0.448, *T*_max_ = 0.754	*k* = −17→10
14856 measured reflections	*l* = −26→23

### Refinement

**Table:**

Refinement on *F*^2^	Primary atom site location: structure-invariant direct methods
Least-squares matrix: full	Secondary atom site location: difference Fourier map
*R*[*F*^2^ > 2σ(*F*^2^)] = 0.048	Hydrogen site location: inferred from neighbouring sites
*wR*(*F*^2^) = 0.128	H-atom parameters constrained
*S* = 1.01	*w* = 1/[σ^2^(*F*_o_^2^) + (0.0594*P*)^2^ + 0.1787*P*] where *P* = (*F*_o_^2^ + 2*F*_c_^2^)/3
3352 reflections	(Δ/σ)_max_ < 0.001
182 parameters	Δρ_max_ = 1.43 e Å^−^^3^
0 restraints	Δρ_min_ = −1.09 e Å^−^^3^

### Special details

**Table:**

Geometry. All e.s.d.'s (except the e.s.d. in the dihedral angle between two l.s. planes) are estimated using the full covariance matrix. The cell e.s.d.'s are taken into account individually in the estimation of e.s.d.'s in distances, angles and torsion angles; correlations between e.s.d.'s in cell parameters are only used when they are defined by crystal symmetry. An approximate (isotropic) treatment of cell e.s.d.'s is used for estimating e.s.d.'s involving l.s. planes.
Refinement. Refinement of *F*^2^ against ALL reflections. The weighted *R*-factor *wR* and goodness of fit *S* are based on *F*^2^, conventional *R*-factors *R* are based on *F*, with *F* set to zero for negative *F*^2^. The threshold expression of *F*^2^ > σ(*F*^2^) is used only for calculating *R*-factors(gt) *etc*. and is not relevant to the choice of reflections for refinement. *R*-factors based on *F*^2^ are statistically about twice as large as those based on *F*, and *R*- factors based on ALL data will be even larger.

### Fractional atomic coordinates and isotropic or equivalent isotropic displacement parameters (Å^2^)

**Table:**

	*x*	*y*	*z*	*U*_iso_*/*U*_eq_	
Br1	0.06086 (10)	0.39989 (3)	0.44439 (2)	0.0693 (2)	
S1	0.41495 (17)	0.01866 (7)	0.27290 (5)	0.0348 (2)	
O1	0.6125 (5)	0.38278 (19)	0.01376 (14)	0.0509 (8)	
O2	0.2589 (5)	0.4559 (2)	0.05304 (15)	0.0528 (8)	
H2A	0.3084	0.5024	0.0293	0.079*	
O3	0.3343 (5)	−0.07285 (18)	0.30378 (14)	0.0459 (7)	
O4	0.6772 (4)	0.0308 (2)	0.25256 (13)	0.0458 (7)	
N1	0.2274 (5)	0.0325 (2)	0.20612 (15)	0.0353 (7)	
H1	0.1085	−0.0116	0.1965	0.042*	
C1	0.1820 (8)	0.2852 (3)	0.3973 (2)	0.0448 (10)	
C2	0.0653 (8)	0.1929 (3)	0.4067 (2)	0.0498 (11)	
H2	−0.0666	0.1862	0.4372	0.060*	
C3	0.1449 (7)	0.1108 (3)	0.3707 (2)	0.0434 (10)	
H3	0.0678	0.0478	0.3770	0.052*	
C4	0.3401 (6)	0.1213 (3)	0.32476 (18)	0.0339 (9)	
C5	0.4571 (8)	0.2152 (3)	0.3160 (2)	0.0472 (10)	
H5	0.5886	0.2222	0.2854	0.057*	
C6	0.3796 (8)	0.2975 (3)	0.3523 (2)	0.0548 (12)	
H6	0.4582	0.3604	0.3468	0.066*	
C7	0.2632 (6)	0.1189 (2)	0.16378 (18)	0.0310 (8)	
C8	0.4623 (7)	0.1184 (3)	0.1193 (2)	0.0399 (9)	
H8	0.5652	0.0608	0.1148	0.048*	
C9	0.5087 (7)	0.2035 (3)	0.08134 (19)	0.0398 (9)	
H9	0.6459	0.2037	0.0521	0.048*	
C10	0.3537 (7)	0.2881 (3)	0.08650 (18)	0.0324 (8)	
C11	0.1471 (7)	0.2863 (3)	0.1297 (2)	0.0412 (10)	
H11	0.0378	0.3424	0.1326	0.049*	
C12	0.1038 (7)	0.2019 (3)	0.16820 (19)	0.0411 (9)	
H12	−0.0340	0.2011	0.1973	0.049*	
C13	0.4143 (7)	0.3801 (3)	0.04823 (18)	0.0372 (9)	

### Atomic displacement parameters (Å^2^)

**Table:**

	*U*^11^	*U*^22^	*U*^33^	*U*^12^	*U*^13^	*U*^23^
Br1	0.0983 (4)	0.0484 (3)	0.0624 (4)	0.0061 (2)	0.0194 (3)	−0.0097 (2)
S1	0.0293 (5)	0.0328 (5)	0.0428 (6)	0.0016 (4)	0.0084 (4)	0.0072 (4)
O1	0.0523 (17)	0.0412 (16)	0.061 (2)	0.0101 (12)	0.0304 (15)	0.0144 (13)
O2	0.0589 (17)	0.0363 (16)	0.065 (2)	0.0145 (14)	0.0293 (15)	0.0186 (14)
O3	0.0485 (16)	0.0331 (15)	0.0567 (19)	0.0037 (12)	0.0135 (13)	0.0141 (13)
O4	0.0271 (13)	0.0532 (18)	0.0579 (19)	0.0035 (11)	0.0117 (12)	0.0064 (13)
N1	0.0321 (16)	0.0332 (17)	0.041 (2)	−0.0093 (12)	0.0037 (14)	0.0058 (14)
C1	0.056 (3)	0.040 (2)	0.039 (2)	0.0045 (19)	0.0048 (19)	−0.0047 (18)
C2	0.053 (3)	0.051 (3)	0.047 (3)	−0.003 (2)	0.021 (2)	0.001 (2)
C3	0.047 (2)	0.037 (2)	0.047 (3)	−0.0076 (17)	0.014 (2)	0.0067 (18)
C4	0.0300 (19)	0.034 (2)	0.038 (2)	−0.0021 (15)	0.0045 (16)	0.0050 (16)
C5	0.048 (2)	0.044 (2)	0.051 (3)	−0.0081 (18)	0.019 (2)	0.001 (2)
C6	0.064 (3)	0.037 (2)	0.064 (3)	−0.014 (2)	0.017 (2)	0.000 (2)
C7	0.0308 (18)	0.0265 (19)	0.036 (2)	−0.0010 (14)	0.0040 (16)	0.0025 (15)
C8	0.044 (2)	0.031 (2)	0.046 (2)	0.0101 (16)	0.0135 (18)	0.0014 (17)
C9	0.043 (2)	0.035 (2)	0.042 (2)	0.0037 (17)	0.0171 (18)	0.0011 (18)
C10	0.0332 (19)	0.030 (2)	0.034 (2)	−0.0001 (15)	0.0058 (16)	0.0006 (16)
C11	0.035 (2)	0.034 (2)	0.056 (3)	0.0119 (15)	0.0147 (18)	0.0088 (19)
C12	0.034 (2)	0.042 (2)	0.048 (3)	0.0069 (16)	0.0162 (18)	0.0083 (19)
C13	0.036 (2)	0.035 (2)	0.040 (2)	−0.0002 (17)	0.0072 (18)	0.0029 (17)

### Geometric parameters (Å, °)

**Table:**

Br1—C1	1.896 (4)	C4—C5	1.388 (5)
S1—O3	1.422 (2)	C5—C6	1.370 (5)
S1—O4	1.427 (2)	C5—H5	0.9300
S1—N1	1.634 (3)	C6—H6	0.9300
S1—C4	1.754 (4)	C7—C12	1.370 (5)
O1—C13	1.247 (4)	C7—C8	1.376 (5)
O2—C13	1.284 (4)	C8—C9	1.379 (5)
O2—H2A	0.8200	C8—H8	0.9300
N1—C7	1.434 (4)	C9—C10	1.374 (5)
N1—H1	0.8600	C9—H9	0.9300
C1—C2	1.371 (5)	C10—C11	1.389 (5)
C1—C6	1.387 (5)	C10—C13	1.472 (5)
C2—C3	1.370 (5)	C11—C12	1.375 (5)
C2—H2	0.9300	C11—H11	0.9300
C3—C4	1.388 (5)	C12—H12	0.9300
C3—H3	0.9300		
			
O3—S1—O4	120.55 (15)	C5—C6—C1	118.8 (4)
O3—S1—N1	106.15 (15)	C5—C6—H6	120.6
O4—S1—N1	106.97 (15)	C1—C6—H6	120.6
O3—S1—C4	108.90 (16)	C12—C7—C8	120.2 (3)
O4—S1—C4	107.95 (16)	C12—C7—N1	120.5 (3)
N1—S1—C4	105.33 (16)	C8—C7—N1	119.3 (3)
C13—O2—H2A	109.5	C7—C8—C9	119.8 (3)
C7—N1—S1	119.3 (2)	C7—C8—H8	120.1
C7—N1—H1	120.4	C9—C8—H8	120.1
S1—N1—H1	120.4	C10—C9—C8	120.5 (3)
C2—C1—C6	121.6 (4)	C10—C9—H9	119.8
C2—C1—Br1	119.1 (3)	C8—C9—H9	119.8
C6—C1—Br1	119.2 (3)	C9—C10—C11	119.2 (3)
C3—C2—C1	119.3 (4)	C9—C10—C13	119.7 (3)
C3—C2—H2	120.3	C11—C10—C13	121.0 (3)
C1—C2—H2	120.3	C12—C11—C10	120.1 (3)
C2—C3—C4	120.2 (3)	C12—C11—H11	119.9
C2—C3—H3	119.9	C10—C11—H11	119.9
C4—C3—H3	119.9	C7—C12—C11	120.1 (3)
C5—C4—C3	119.8 (3)	C7—C12—H12	119.9
C5—C4—S1	120.6 (3)	C11—C12—H12	119.9
C3—C4—S1	119.4 (3)	O1—C13—O2	122.6 (3)
C6—C5—C4	120.3 (4)	O1—C13—C10	120.0 (3)
C6—C5—H5	119.8	O2—C13—C10	117.4 (3)
C4—C5—H5	119.8		
			
O3—S1—N1—C7	−179.7 (2)	Br1—C1—C6—C5	−176.8 (3)
O4—S1—N1—C7	−49.8 (3)	S1—N1—C7—C12	−99.7 (4)
C4—S1—N1—C7	64.9 (3)	S1—N1—C7—C8	79.3 (4)
C6—C1—C2—C3	−0.2 (7)	C12—C7—C8—C9	3.0 (6)
Br1—C1—C2—C3	177.3 (3)	N1—C7—C8—C9	−175.9 (3)
C1—C2—C3—C4	−0.5 (6)	C7—C8—C9—C10	−1.5 (6)
C2—C3—C4—C5	0.8 (6)	C8—C9—C10—C11	−0.9 (6)
C2—C3—C4—S1	−173.2 (3)	C8—C9—C10—C13	176.9 (4)
O3—S1—C4—C5	162.7 (3)	C9—C10—C11—C12	1.9 (6)
O4—S1—C4—C5	30.2 (4)	C13—C10—C11—C12	−175.8 (4)
N1—S1—C4—C5	−83.8 (3)	C8—C7—C12—C11	−2.0 (6)
O3—S1—C4—C3	−23.2 (3)	N1—C7—C12—C11	176.9 (3)
O4—S1—C4—C3	−155.7 (3)	C10—C11—C12—C7	−0.5 (6)
N1—S1—C4—C3	90.3 (3)	C9—C10—C13—O1	−3.4 (6)
C3—C4—C5—C6	−0.3 (6)	C11—C10—C13—O1	174.4 (3)
S1—C4—C5—C6	173.7 (3)	C9—C10—C13—O2	177.8 (4)
C4—C5—C6—C1	−0.4 (6)	C11—C10—C13—O2	−4.5 (5)
C2—C1—C6—C5	0.7 (7)		

### Hydrogen-bond geometry (Å, °)

**Table:**

*D*—H···*A*	*D*—H	H···*A*	*D*···*A*	*D*—H···*A*
O2—H2A···O1^i^	0.82	1.80	2.606 (4)	171
N1—H1···O4^ii^	0.86	2.57	3.001 (3)	112
C2—H2···O1^iii^	0.93	2.46	3.361 (5)	164
C3—H3···O2^iv^	0.93	2.53	3.314 (5)	143
C11—H11···O3^v^	0.93	2.58	3.395 (5)	146

Symmetry codes: (i) −*x*+1, −*y*+1, −*z*; (ii) *x*−1, *y*, *z*; (iii) *x*−1, −*y*+1/2, *z*+1/2; (iv) −*x*, *y*−1/2, −*z*+1/2; (v) −*x*, *y*+1/2, −*z*+1/2.
